# Supplementary material for: The second will be first: competition on directed networks
Source: Sci Rep. 2016 Jun 7;6:27116. doi: 10.1038/srep27116 (PMC4895223; doi:10.1038/srep27116)
Supplement: Supplementary Information [file srep27116-s1.pdf]

,

Supplementary Information – The second will be first: competition on directed networks

Giulia Cencetti,[1,2] Franco Bagnoli,[3,2] Francesca Di Patti,[3,2] and Duccio Fanelli[3,2,*]

[1]Dipartimento di Ingegneria dell'Informazione and CSDC, Università degli Studi di Firenze, via S. Marta 3I-50139 Firenze, Italy

[2]INFN, Sezione di Firenze, Florence, Italy

[3]Dipartimento di Fisica e Astronomia and CSDC, Università degli Studi di Firenze, via G. Sansone 1, 50019 Sesto Fiorentino, Italia

PACS numbers: 89.75.Hc 89.75.Kd 89.75.Fb

In the video annexed as Supplementary Information we display the density plot for (V_{ij} vs. σ_{ij}), as obtained for increasing values of the rewiring probability p. A detail description of the aforementioned quantities is provided in the main body of the paper.

* Correspondence to `duccio.fanelli@unifi.it`
